# Supplementary material for: Unraveling the glycosylated immunopeptidome with HLA-Glyco
Source: Nat Commun. 2023 Jun 12;14:3461. doi: 10.1038/s41467-023-39270-2 (PMC10258777; doi:10.1038/s41467-023-39270-2)
Supplement: Supplementary file 1 — Supplementary Information [file 41467_2023_39270_MOESM1_ESM.pdf]

Supplementary materials

Contents

Supplementary Figure 1 2
Supplementary Figure 2 3
Supplementary Figure 3 12
Supplementary Note 1 13
Supplementary Note 2 15

Supplementary Figure 1

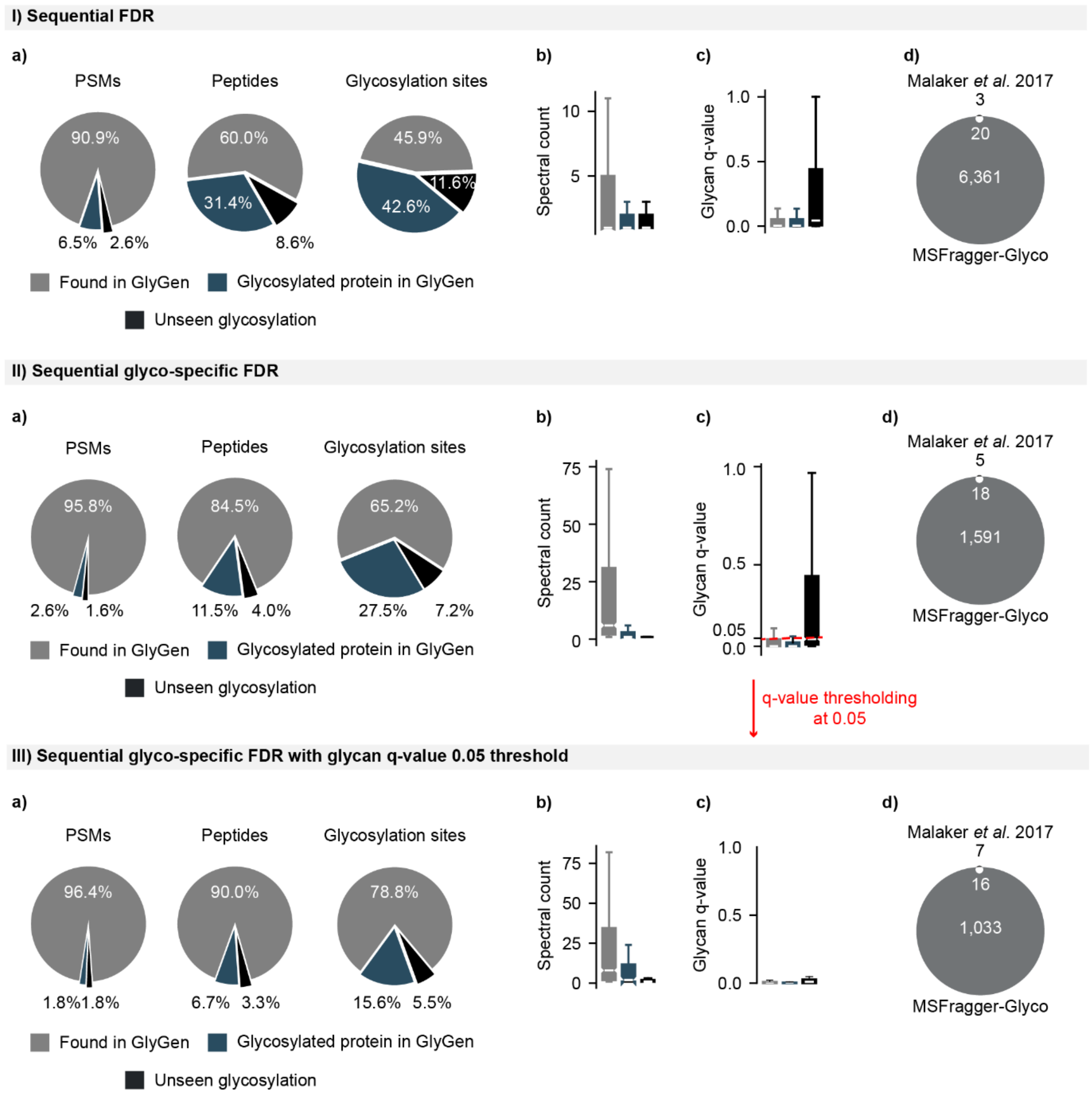

Supplementary Figure 1: comparison of three FDR control strategies for HLA-glycosylated peptides. Strategy I (sequential FDR) for enzymatic searches used in glyco-proteomics, **Strategy II** (sequential glyco-specific FDR) for non-specific searches developed in this study, and **Strategy III** (Strategy II + glycan q-value threshold of 0.05) used in this study. **a)** Percentage of glyco-PSMs, glycopeptides, and glycosylation sites in GlyGen. **b)** Abundance of the three categories. **c)** Glycan q-value range. **d)** Comparison of glycosylation sites with another study (Malaker *et al.* 2017). Boxplot values are provided in the subsequent text with the following order: Bottom whisker - Q1 - Median - Q3 - Interquartile range - Top whisker - Minimum - Maximum. **Ib)** Grey boxplot values: 1-1-1-5-4-11-1-13700. Blue boxplot values: 1-1-1-2-1-3-1-335. Black boxplot values: 1-1-1-2-1-3-1-501. **Ic)** Grey boxplot values: 0-0.0002-0.0021-0.0544-0.0542-0.1356-0-1. Blue boxplot values: 0-0.0003-0.0033-0.0542-0.0539-0.1348-0-1. Black boxplot values: 0-0.0049-0.0429-0.4403-0.4354-1-0-1. **IIb)** Grey boxplot values: 1-2-6-31-29-74-1-10302. Blue boxplot values: 1-1-1-3-2-6-1-258. Black boxplot values: 1-1-1-1-0-1-1-384. **IIc)** Grey boxplot values: 0-0-0.0011-0.04-0.04-0.1-0-1. Blue boxplot values: 0-0-0.0008-0.0219-0.0219-0.0546-0-1. Black boxplot values: 0-0.0089-0.04-0.3915-0.3826-0.9651-0-1. **IIIb)** Grey boxplot values: 1-2-8-34.5-32.5-82-1-5370. Blue boxplot values: 1-1-2-11.75-10.75-24-1-251. Black boxplot values: 1-1-1-2-1-3-1-215. **IIIc)** Grey boxplot values: 0-0-0.0003-0.0088-0.0088-0.0221-0-0.0500. Blue boxplot values: 0-0-0.0003-0.0038-0.0038-0.0095-0-0.0481. Black boxplot values: 0-0.0003-0.0094-0.0274-0.0271-0.047-0-0.047.

Supplementary Figure 2

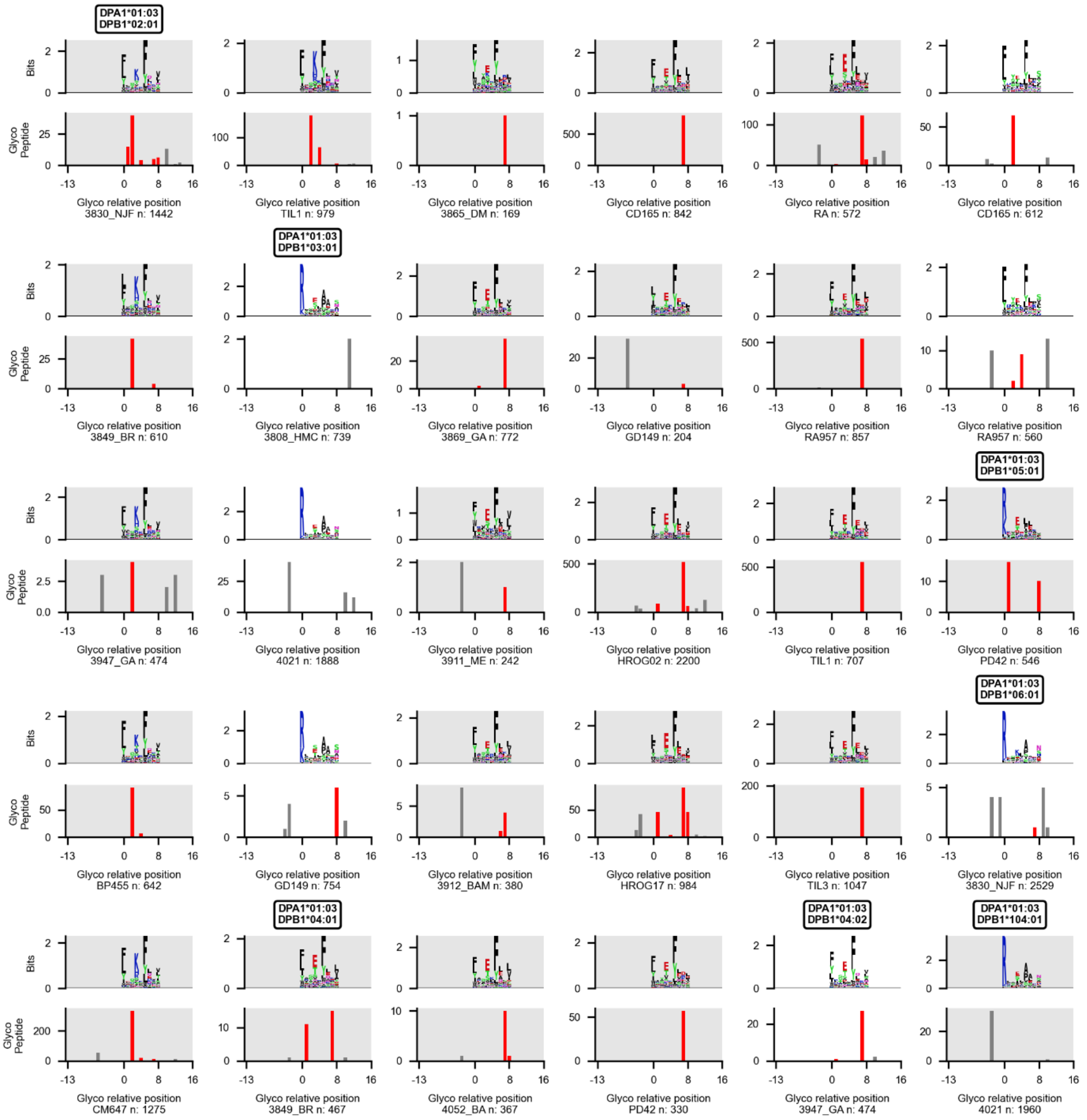

Supplementary Figure 2 – part 1: A visual over glycosylation with reference to the HLA class II binding core after a fully unsupervised deconvolution of all analyzed samples. HLA-binding cores for all analyzed samples are shown per allele in a column-wise fashion. The HLA motif signature is shown (on top) for all peptides of a particular sample passing a percentile rank threshold of 20 after NetMHCIIpan 4.1 binding affinity prediction. For samples with multiple HLA class II alleles, the peptides were assigned to the allele with the lowest percentile rank value. The number of glycosylated peptides per position relative to the HLA-binding core is shown at the bottom of the HLA motif signature. Negative values refer to glycosylation positions upstream of the HLA-binding core; values between 0 and 8 represent positions within the HLA-binding core; and values ≥ 9 refer to positions downstream of the HLA-binding core. Glycosylation within the HLA-binding core is shown in red, whereas glycosylation upstream and downstream is shown in gray.

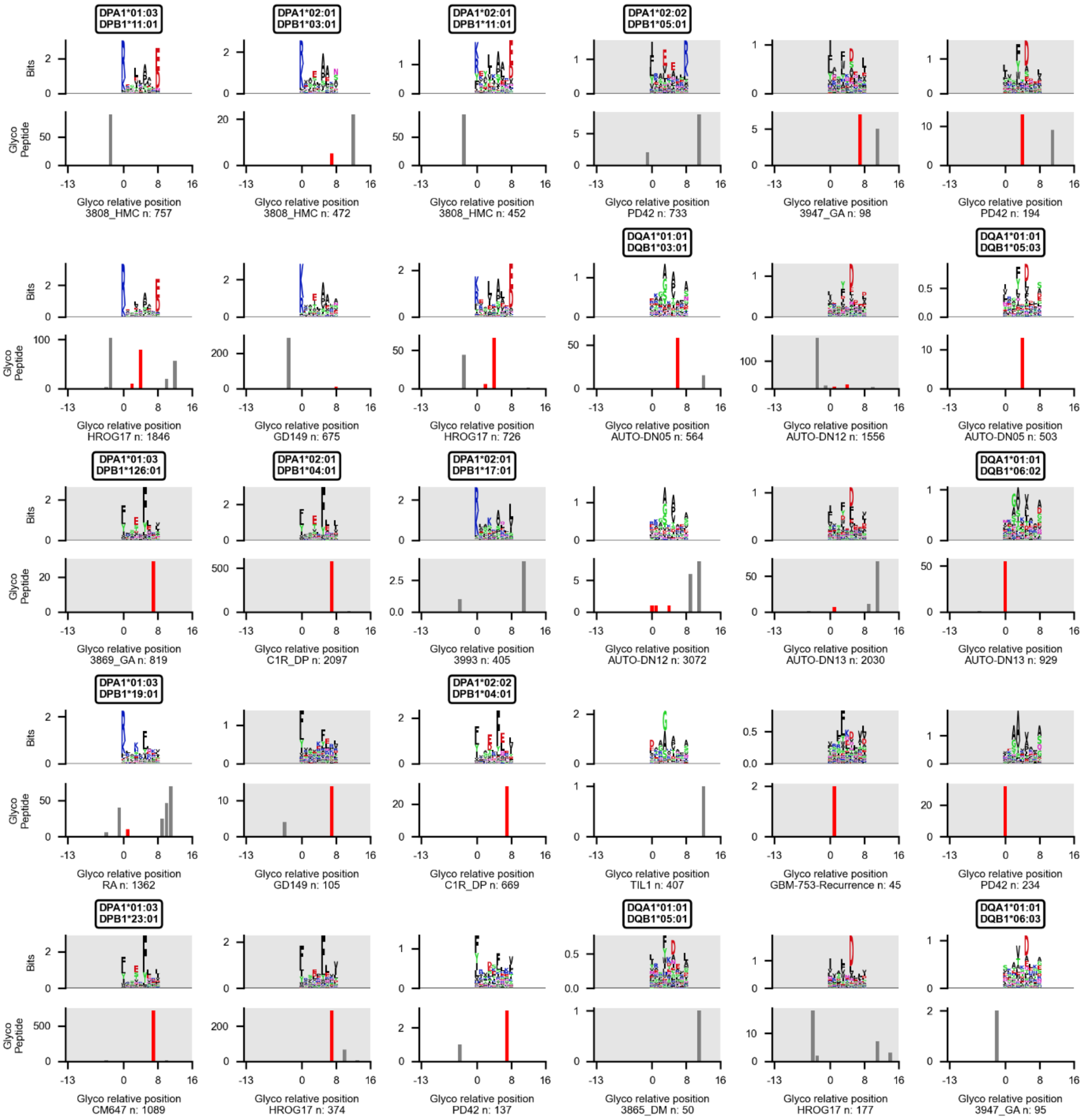

Supplementary Figure 2 – part 2: HLA-binding cores for all analyzed samples are shown per allele in a column-wise fashion. The HLA motif signature is shown (on top) for all peptides of a particular sample passing a percentile rank threshold of 20 after NetMHCIIpan 4.1 binding affinity prediction. For samples with multiple HLA class II alleles, the peptides were assigned to the allele with the lowest percentile rank value. The number of glycosylated peptides per position relative to the HLA-binding core is shown at the bottom of the HLA motif signature. Negative values refer to glycosylation positions upstream of the HLA-binding core; values between 0 and 8 represent positions within the HLA-binding core; and values ≥ 9 refer to positions downstream of the HLA-binding core. Glycosylation within the HLA-binding core is shown in red, whereas glycosylation upstream and downstream is shown in gray.

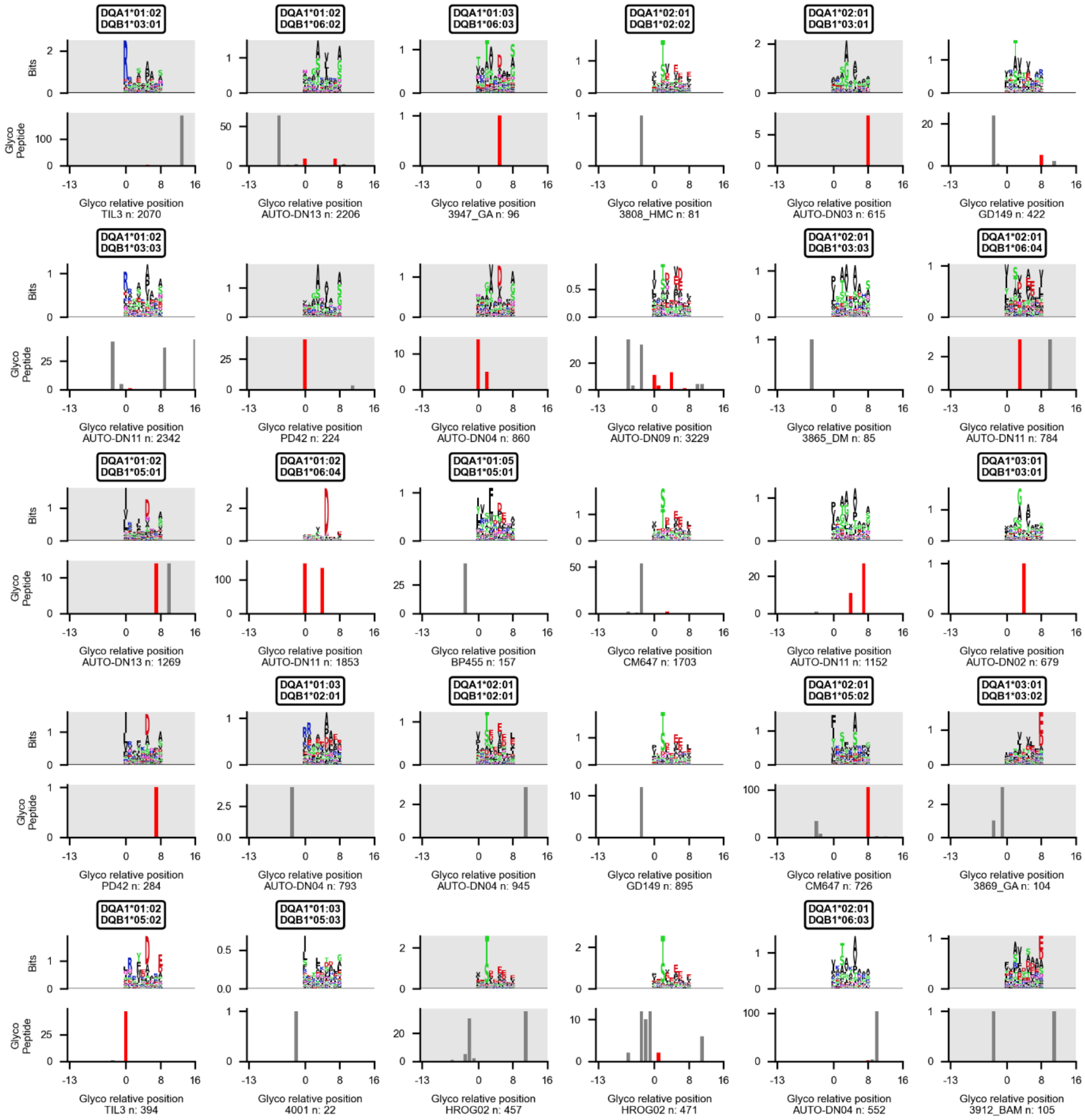

Supplementary Figure 2 – part 3: HLA-binding cores for all analyzed samples are shown per allele in a column-wise fashion. HLA-binding cores for all analyzed samples are shown per allele in a column-wise fashion. The HLA motif signature is shown (on top) for all peptides of a particular sample passing a percentile rank threshold of 20 after NetMHCIIpan 4.1 binding affinity prediction. For samples with multiple HLA class II alleles, the peptides were assigned to the allele with the lowest percentile rank value. The number of glycosylated peptides per position relative to the HLA-binding core is shown at the bottom of the HLA motif signature. Negative values refer to glycosylation positions upstream of the HLA-binding core; values between 0 and 8 represent positions within the HLA-binding core; and values ≥ 9 refer to positions downstream of the HLA-binding core. Glycosylation within the HLA-binding core is shown in red, whereas glycosylation upstream and downstream is shown in gray.

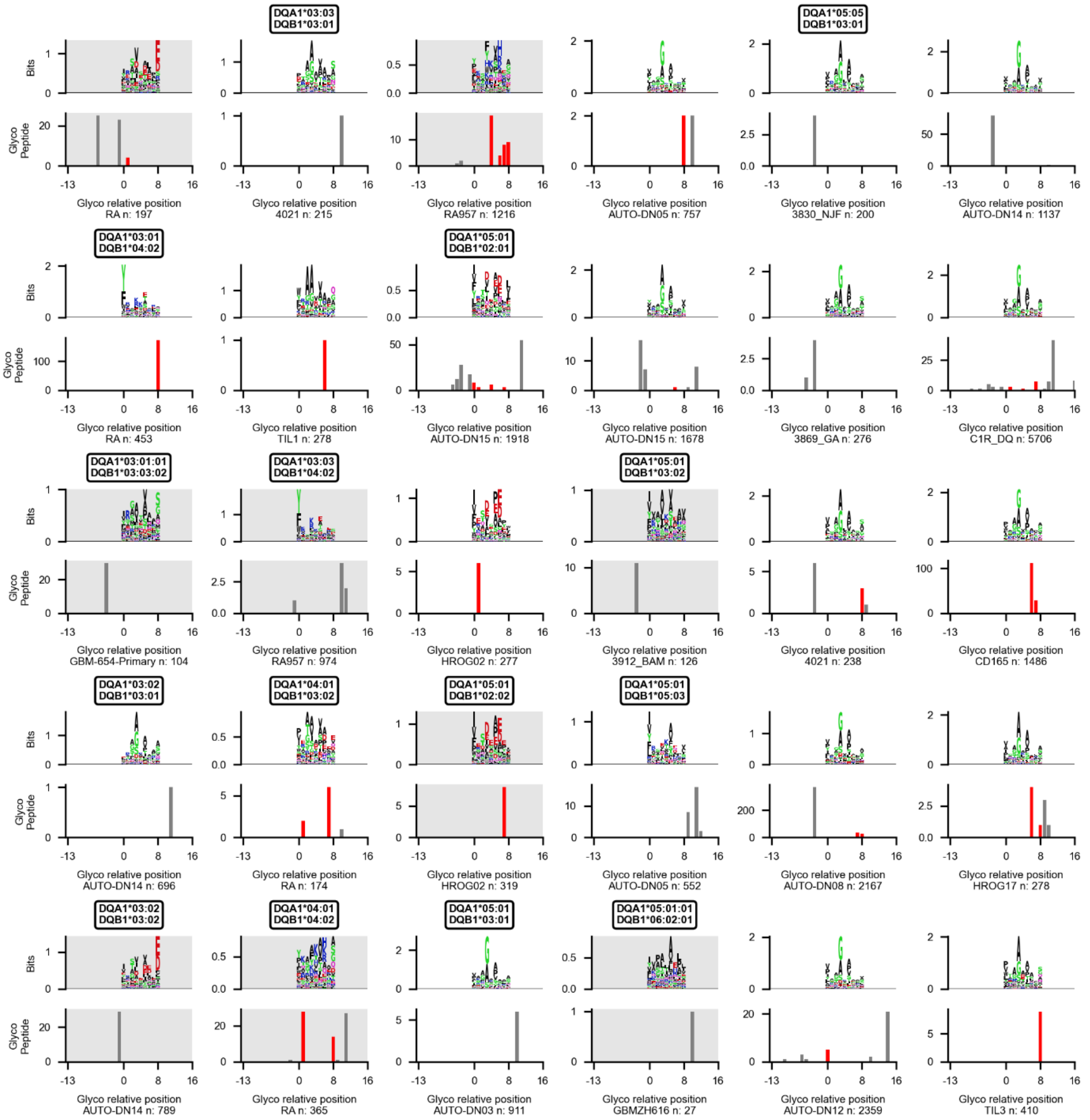

Supplementary Figure 2 – part 4: HLA-binding cores for all analyzed samples are shown per allele in a column-wise fashion. HLA-binding cores for all analyzed samples are shown per allele in a column-wise fashion. The HLA motif signature is shown (on top) for all peptides of a particular sample passing a percentile rank threshold of 20 after NetMHCIIpan 4.1 binding affinity prediction. For samples with multiple HLA class II alleles, the peptides were assigned to the allele with the lowest percentile rank value. The number of glycosylated peptides per position relative to the HLA-binding core is shown at the bottom of the HLA motif signature. Negative values refer to glycosylation positions upstream of the HLA-binding core; values between 0 and 8 represent positions within the HLA-binding core; and values ≥ 9 refer to positions downstream of the HLA-binding core. Glycosylation within the HLA-binding core is shown in red, whereas glycosylation upstream and downstream is shown in gray.

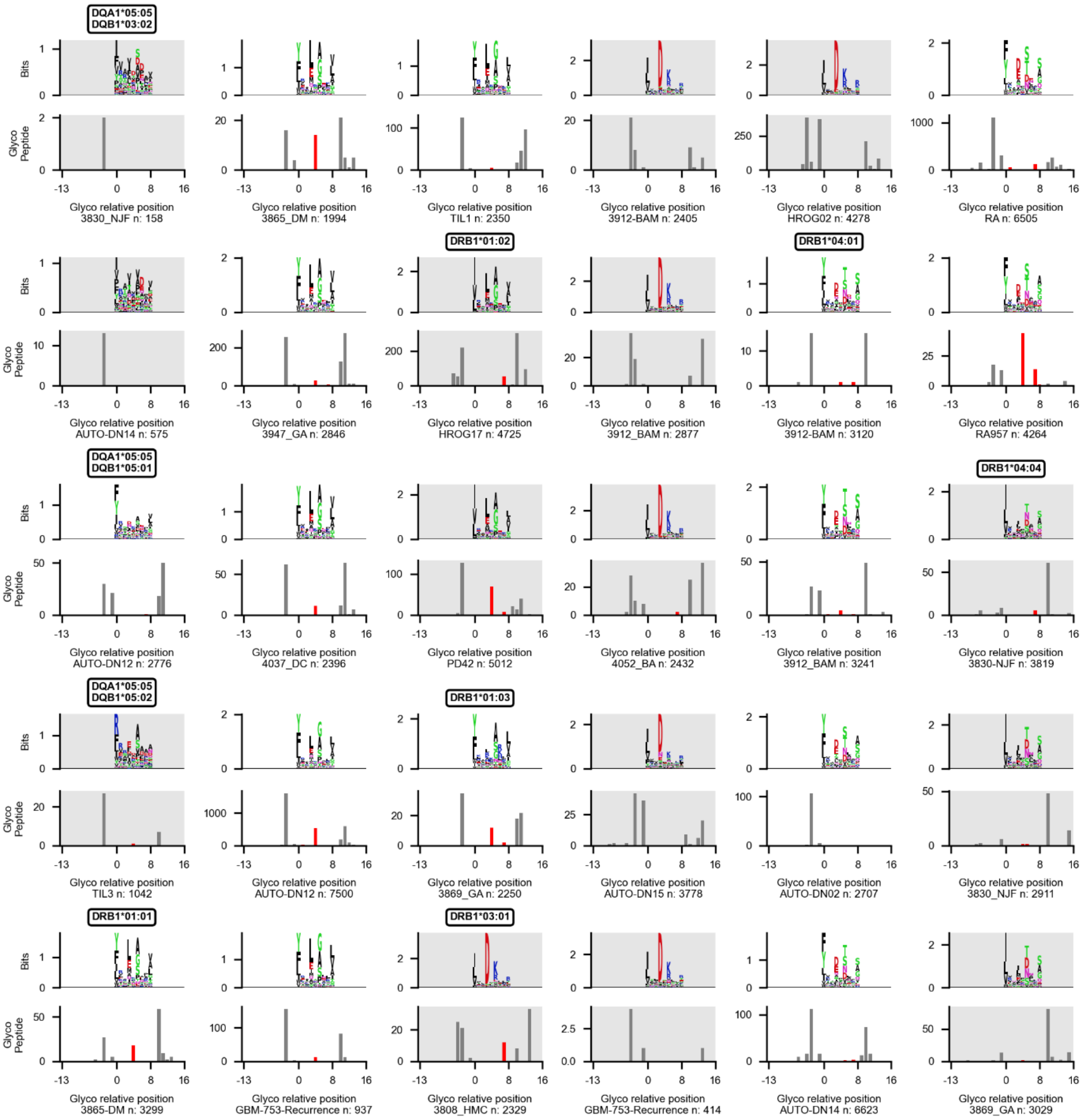

Supplementary Figure 2 – part 5: HLA-binding cores for all analyzed samples are shown per allele in a column-wise fashion. HLA-binding cores for all analyzed samples are shown per allele in a column-wise fashion. The HLA motif signature is shown (on top) for all peptides of a particular sample passing a percentile rank threshold of 20 after NetMHCIIpan 4.1 binding affinity prediction. For samples with multiple HLA class II alleles, the peptides were assigned to the allele with the lowest percentile rank value. The number of glycosylated peptides per position relative to the HLA-binding core is shown at the bottom of the HLA motif signature. Negative values refer to glycosylation positions upstream of the HLA-binding core; values between 0 and 8 represent positions within the HLA-binding core; and values ≥ 9 refer to positions downstream of the HLA-binding core. Glycosylation within the HLA-binding core is shown in red, whereas glycosylation upstream and downstream is shown in gray.

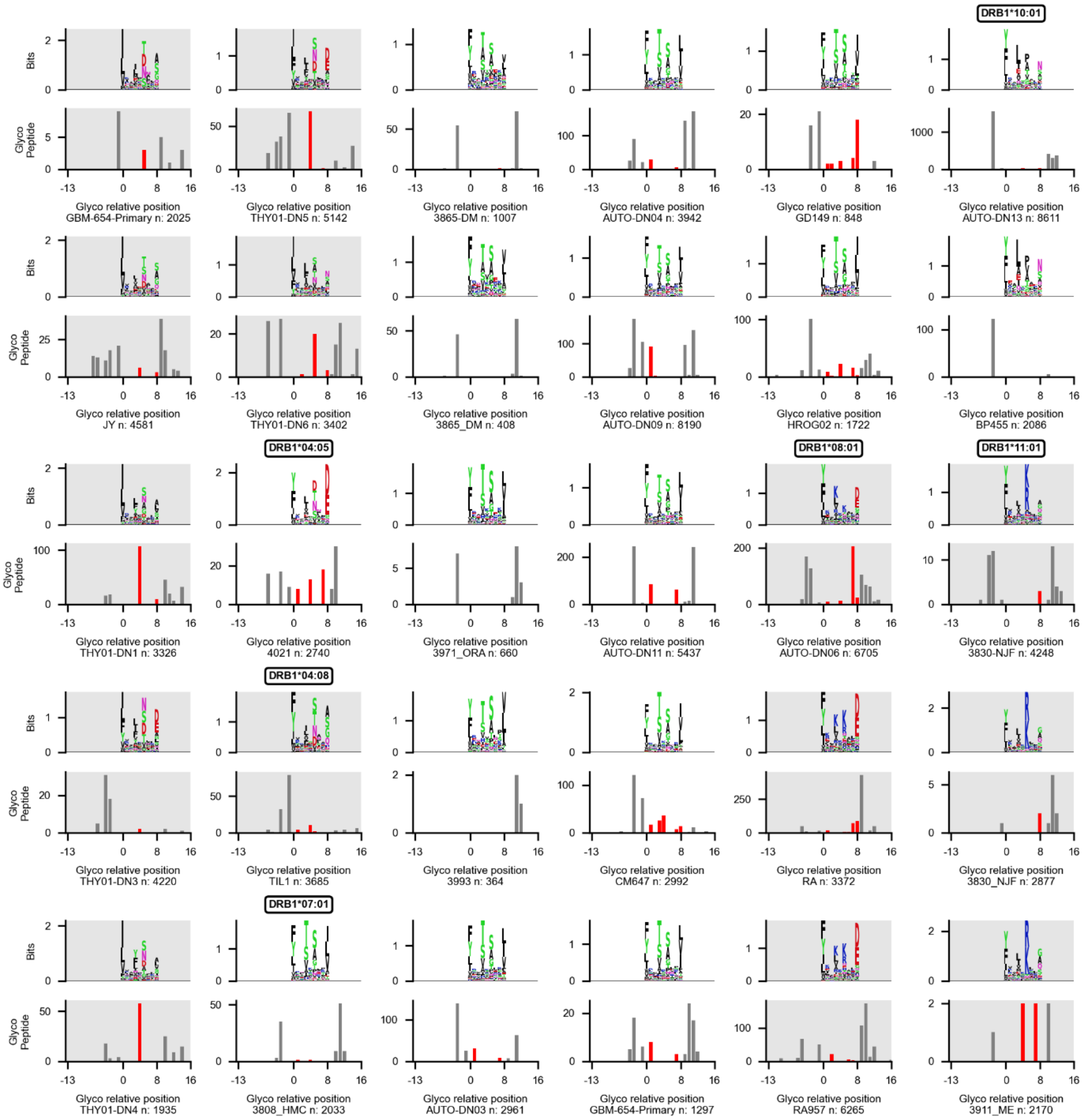

Supplementary Figure 2 – part 6: HLA-binding cores for all analyzed samples are shown per allele in a column-wise fashion. HLA-binding cores for all analyzed samples are shown per allele in a column-wise fashion. The HLA motif signature is shown (on top) for all peptides of a particular sample passing a percentile rank threshold of 20 after NetMHCIIpan 4.1 binding affinity prediction. For samples with multiple HLA class II alleles, the peptides were assigned to the allele with the lowest percentile rank value. The number of glycosylated peptides per position relative to the HLA-binding core is shown at the bottom of the HLA motif signature. Negative values refer to glycosylation positions upstream of the HLA-binding core; values between 0 and 8 represent positions within the HLA-binding core; and values ≥ 9 refer to positions downstream of the HLA-binding core. Glycosylation within the HLA-binding core is shown in red, whereas glycosylation upstream and downstream is shown in gray.

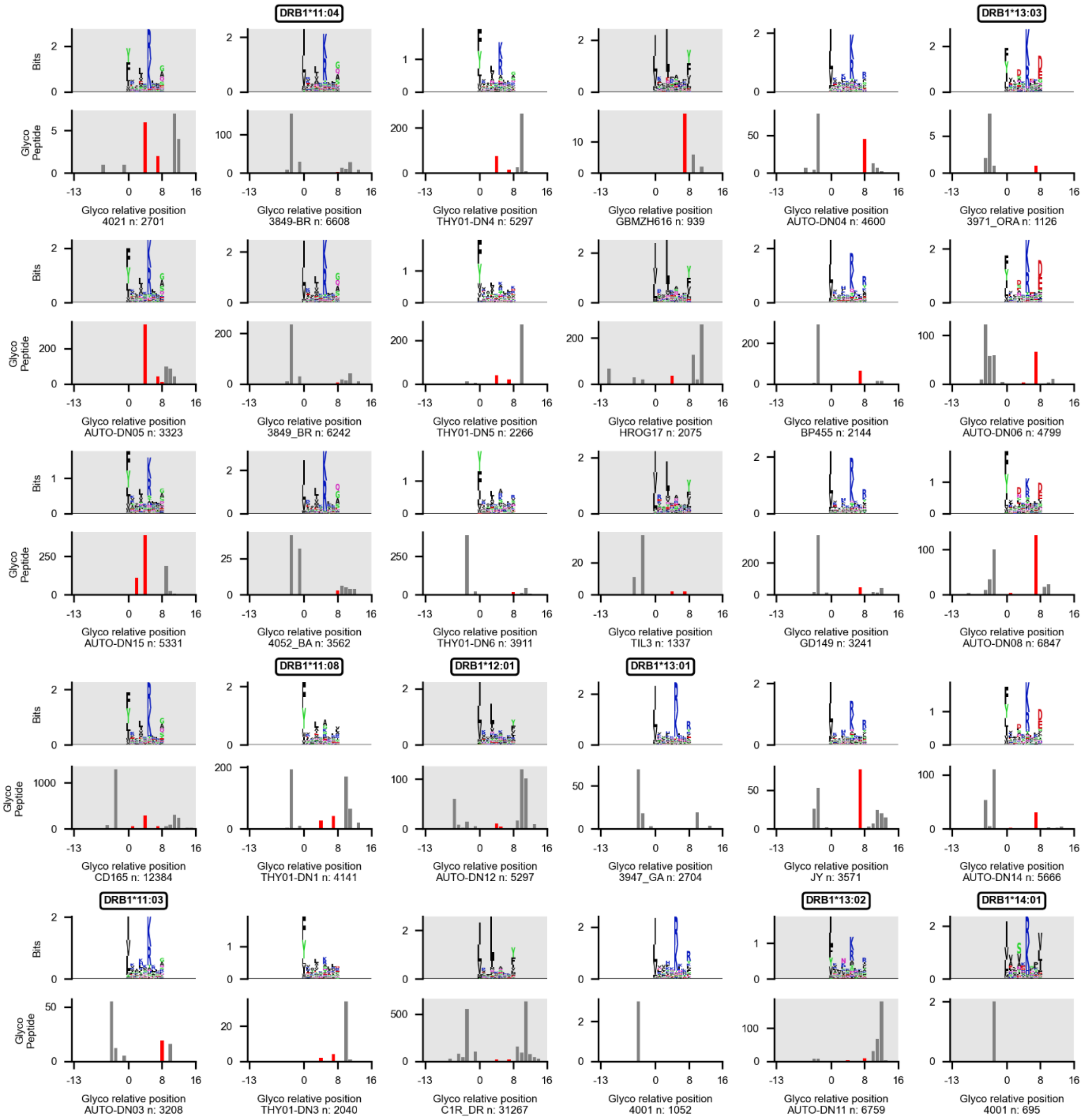

Supplementary Figure 2 – part 7: HLA-binding cores for all analyzed samples are shown per allele in a column-wise fashion. HLA-binding cores for all analyzed samples are shown per allele in a column-wise fashion. The HLA motif signature is shown (on top) for all peptides of a particular sample passing a percentile rank threshold of 20 after NetMHCIIpan 4.1 binding affinity prediction. For samples with multiple HLA class II alleles, the peptides were assigned to the allele with the lowest percentile rank value. The number of glycosylated peptides per position relative to the HLA-binding core is shown at the bottom of the HLA motif signature. Negative values refer to glycosylation positions upstream of the HLA-binding core; values between 0 and 8 represent positions within the HLA-binding core; and values ≥ 9 refer to positions downstream of the HLA-binding core. Glycosylation within the HLA-binding core is shown in red, whereas glycosylation upstream and downstream is shown in gray.

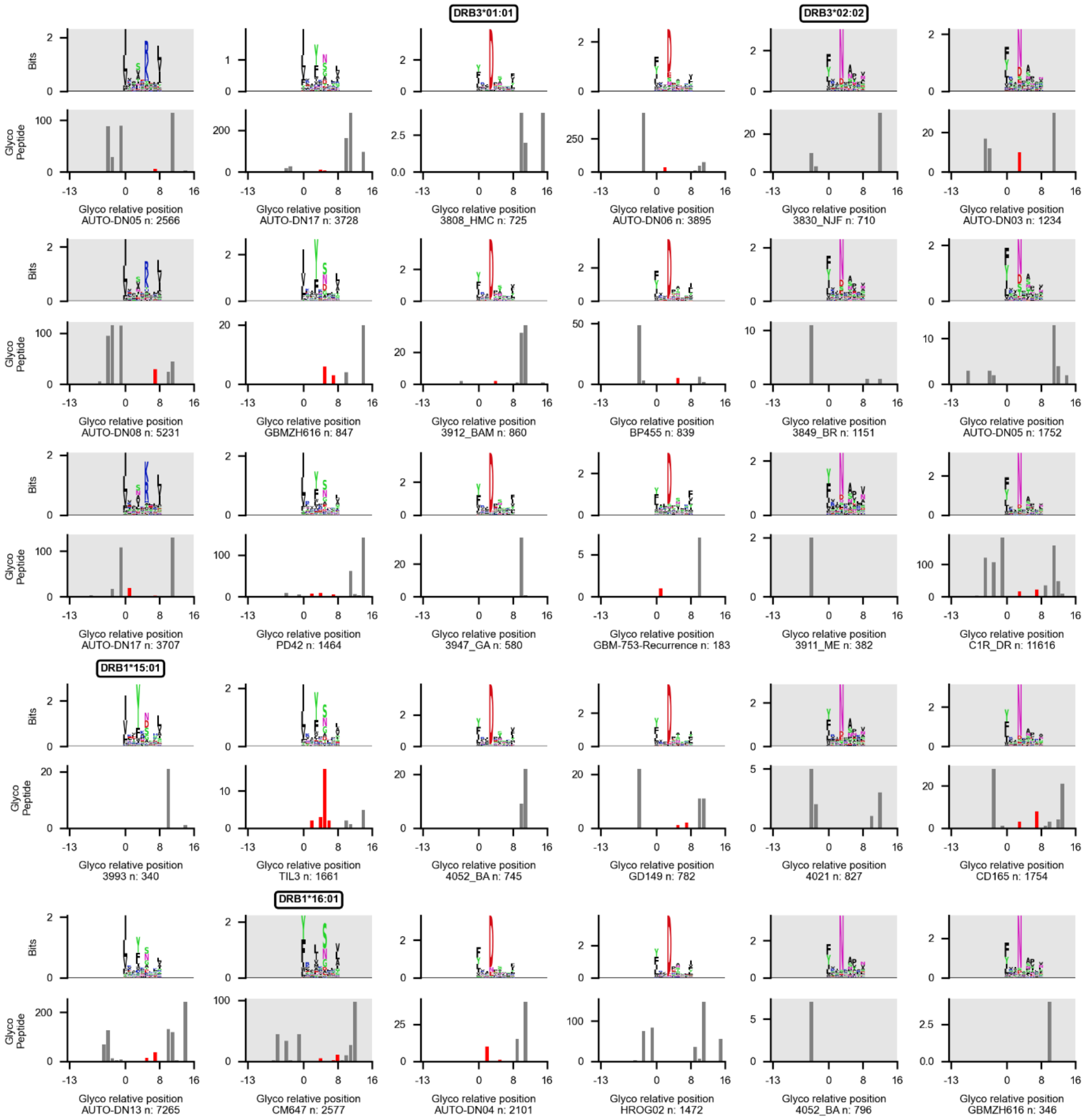

Supplementary Figure 2 – part 8: HLA-binding cores for all analyzed samples are shown per allele in a column-wise fashion. HLA-binding cores for all analyzed samples are shown per allele in a column-wise fashion. The HLA motif signature is shown (on top) for all peptides of a particular sample passing a percentile rank threshold of 20 after NetMHCIIpan 4.1 binding affinity prediction. For samples with multiple HLA class II alleles, the peptides were assigned to the allele with the lowest percentile rank value. The number of glycosylated peptides per position relative to the HLA-binding core is shown at the bottom of the HLA motif signature. Negative values refer to glycosylation positions upstream of the HLA-binding core; values between 0 and 8 represent positions within the HLA-binding core; and values ≥ 9 refer to positions downstream of the HLA-binding core. Glycosylation within the HLA-binding core is shown in red, whereas glycosylation upstream and downstream is shown in gray.

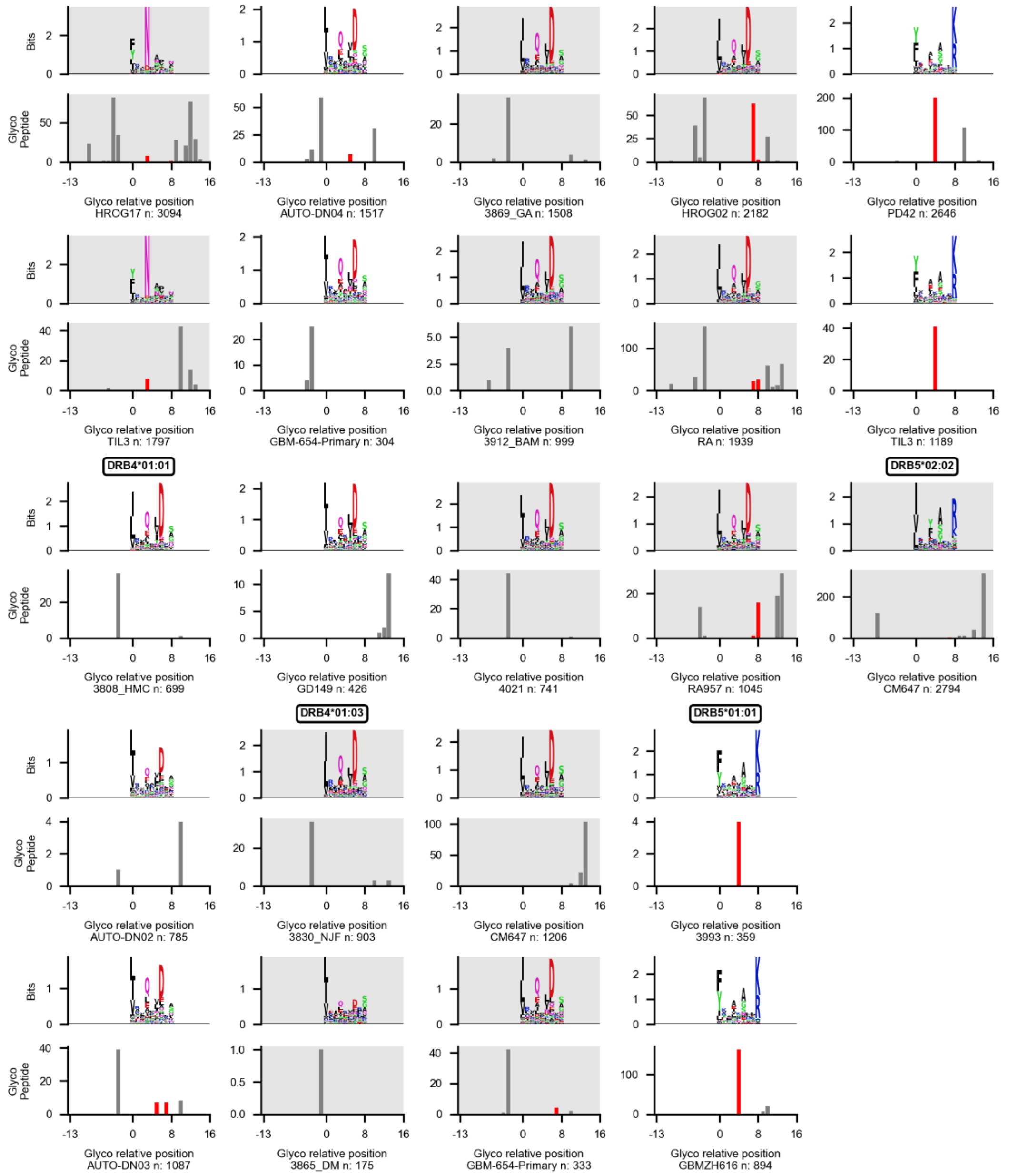

Supplementary Figure 2 – part 9: HLA-binding cores for all analyzed samples are shown per allele in a column-wise fashion. HLA-binding cores for all analyzed samples are shown per allele in a column-wise fashion. The HLA motif signature is shown (on top) for all peptides of a particular sample passing a percentile rank threshold of 20 after NetMHCIIpan 4.1 binding affinity prediction. For samples with multiple HLA class II alleles, the peptides were assigned to the allele with the lowest percentile rank value. The number of glycosylated peptides per position relative to the HLA-binding core is shown at the bottom of the HLA motif signature. Negative values refer to glycosylation positions upstream of the HLA-binding core; values between 0 and 8 represent positions within the HLA-binding core; and values ≥ 9 refer to positions downstream of the HLA-binding core. Glycosylation within the HLA-binding core is shown in red, whereas glycosylation upstream and downstream is shown in gray.

Supplementary Figure 3

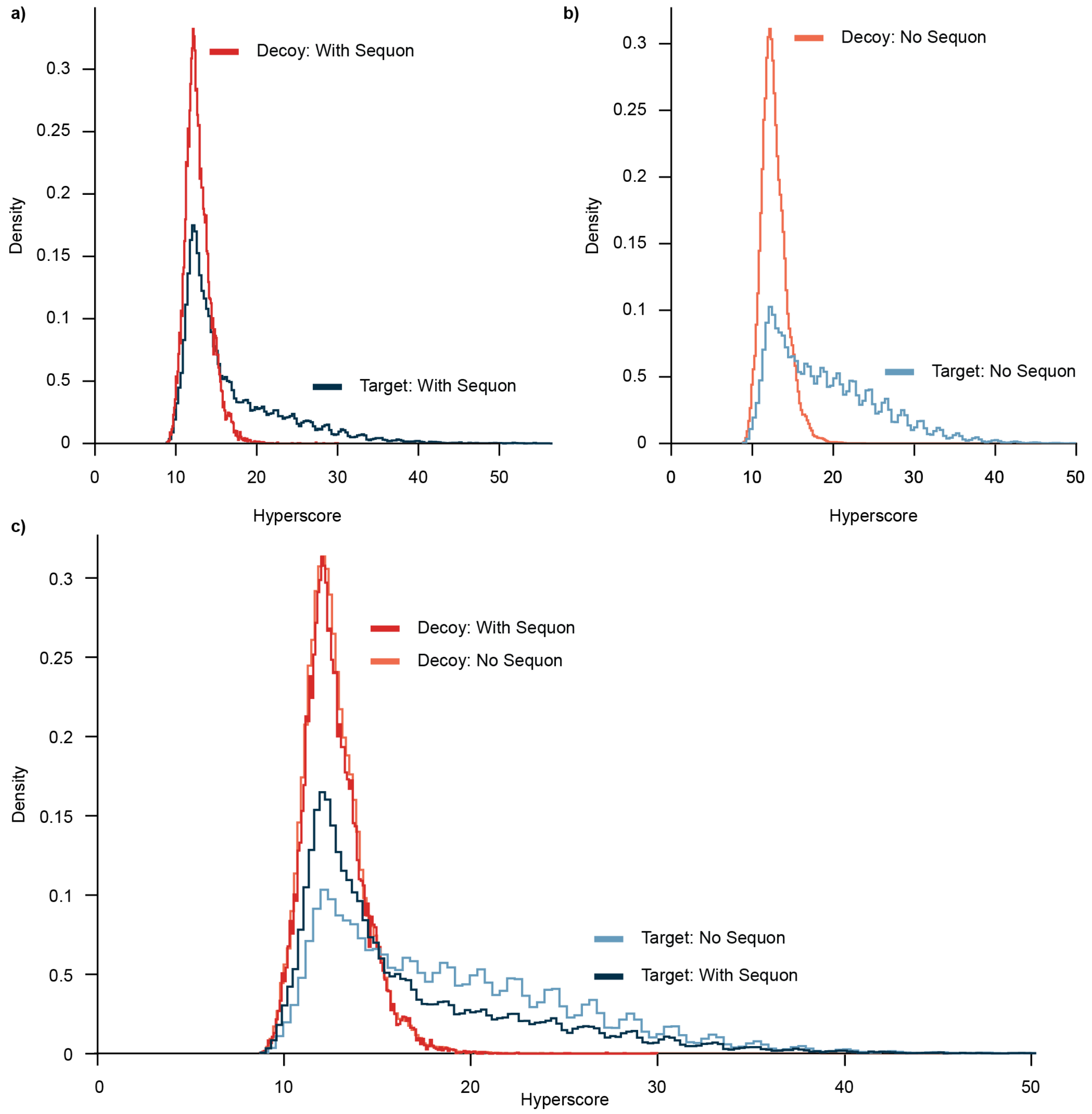

Supplementary Figure 3: Analysis of Hyperscores in Peptides with and without a Sequon, Comparing Target and Decoy Distributions. a) Histogram of Hyperscores for Targets and Decoys in Peptides Containing a Sequon (N-X-S/T). **b)** Histogram of Hyperscores for Targets and Decoys in Peptides Not Containing a Sequon. **c)** Overlaid Histograms of Hyperscores for Targets and Decoys in Peptides with and without a Sequon. It is noteworthy that the distributions of decoys and low-scoring targets exhibit remarkable similarity between peptides with and without sequons.

Supplementary Note 1

The datasets were selected based on the inclusion of studies from PRIDE using the following keywords:

Filter pride datasets based on the following keywords (selected by screening all 17K pride keywords): "Immunoprecipitation", "Immunopeptidome", "Peptidomics", "Affinity purification", "Mhc", "Peptidome", "Hla", "Immunopeptidomics", "Mhc class i", "Ip", "Hla peptidome", "Hla-b*27", "Hla class ii", "Neoantigens", "Immunoinformatics", "Hla-c", "Mhc class 1 ligands", "Proteogenomic cryptic mhc lc-msms maps", "Mhc class i antigen presentation pathway", "Mhc-i peptides", "Mhc i", "Immunopeptidome; hla; lc-ms/ms; netmhcpan; binding prediction", "Mhc ii", "Mhc-i peptide-loading complex", "Mhc affinity prediction", "Mhc-ii peptidomics", "Mhc ligandome", "Mhc i-associated peptides", "Mhc-i", "Mhc class ii", "Antigen presentation/ mhc class ii/ immunopeptidome/ peptide editing/ polymorphism", "Mhc-i peptidomics", "Shotgun proteomics; immunoprecipitation; meiosis; conserved proteins; meioc; ", "Anti-ha immunopurification", "Immunopeptidome; hla; lc-ms/ms; netmhcpan; binding prediction", "Personalized immunotherapy", "Immunoprecipation", "Immunoprecipiation", "Immunoaffinity purification", "Immunoprepicipitations", "Immunopurification", "Antigen presentation/ mhc class ii/ immunopeptidome/ peptide editing/ polymorphism", "Hla-ii", "Hla peptides", "Hla-e", "Hla-b*51", "Hla class i peptides", "Ducaf; hla-drb1*03:01", "Hla typing", "Hla-g", 'Hla class ı ligandome; hla class ı peptide ligands; high ph reversed phase; strong "cation exchange; pre-fractionation', "Hla-b40", "Hla binding motifs", "Hla-dm", "Hla-b27", "Immunopeptidome; hla; lc-ms/ms; netmhcpan; binding prediction", "Hla-b*58:01", "Hla-b*40:02 peptidome", "Hla-dr peptides", "Hla-dr", "Hla-a", "Hla-b57", "Hla class i", "Hla-i", "Hla-a2", "Hla-b", "Interferon gamma; proteomic analysis; hla class i; apm", "Hla-i peptides", "Hla-ligand", "Hla-b*57:03", "Hla-ligandomics", "Hla-a*29:02", "Hla-dr15", "Hla-class i", "Hla-restricted peptide", "Mhc class ii","T cell responses","Hla-b07","Immunopeptidomics","Mhc","Hla class i","Immunoprecipitation","Adaptive immunity","Immunopeptidome","Mhc class i associated peptides","Mhc class ii associated peptides","Macrophages","Immune response","Hla-i","Hla-dr","Hla-dp","Hla-b27; disease association; subtypes","Hla","Hla-b*27","Glycopeptides","Mhcii","Tumor antigens","Peptide spliicng","Antigen processing","Class ii","Dp","Dq","Immunoprecipitation-ms","Homo sapiens","Mhc class i","Mhc binding motifs","Spliced peptides","Mhc-i","Non-canonical peptides","Mhc-ii peptidome","Mhc immunoprecipitation","Hla-b*57:01","Cell surface antigens","Hla binding motifs","Hla binding peptides","Hla class i ligandome","Hla class i peptide ligands; colorectal cancer; tumor heterogeneity; patient-derived organoids; mass spectrometry; affinity

proteomics","Hla class i peptides","Hla class ii","Hla class ii ligandome","Hla class ı ligandome; hla class ı peptide ligands; high ph reversed phase; strong cation exchange; pre-fractionation","Hla class-i","Hla cross-restriction","Hla immunopeptidome","Hla ligandome","Hla ligandomics","Hla peptide","Hla peptides","Hla peptidome","Hla peptidomics","Hla peptidomie","Hla-b*40:02 peptidome","Hla-b*51","Hla-dr-derived self-peptides","Hla-dr15 molecules","Hla-ii","Hla-ligand","Hla-ligandomics","Hla-loh","Hla-restricted peptide","Mhc affinity prediction","Mhc bound peptides","Mhc class i, random peptide library, lc-ms/ms, de novo sequencing","Mhc ii presentation","Mhc immunoaffinity chromatography","Mhc ligandome","Mhc-associated peptide proteomics (mapps)","Mhc-i peptides","Mhc-i peptidome","Mhc-i peptidomics","Mhc-ii","Mhc-ii peptidomics","Immuno precipitation"

Supplementary Note 2

We conducted two entrapment searches to empirically validate the effectiveness of our FDR control methods. Here are the details of each search:

For the first entrapment search, we utilized the raw data from Racle *et al.* 2019 (PXD012308), consisting of 131 raw files. The search was performed against a combined proteome of Human (20,464 sequences) and Arabidopsis thaliana (27,618 sequences). The search settings remained identical to the main HLA-glyco searches. Overall, a total of 916,339 human PSMs and 301 A. thaliana PSMs were identified (yielding a non-glyco entrapment rate of 0.03%). Among these identifications, 19,630 human glycoPSMs were identified, while no A. thaliana glycoPSMs were reported (resulting in a glyco entrapment rate of 0%).

In the second entrapment search, we used data from Liu *et al.* 2017 (PXD005565), which included 3 raw files of yeast enriched N-glycopeptide data. These files were searched alongside 3 raw files from Racle *et al.* 2019 using a human proteome database and the same search settings as our main HLA-glyco method. From the human files, a total of 32,032 PSMs were identified, along with 142 PSMs from the yeast files (leading to a non-glyco entrapment rate of 0.44%). Additionally, 472 glycoPSMs were identified from the human files, whereas no glycoPSMs were found in the yeast files (resulting in a glyco entrapment rate of 0%).

These entrapment searches demonstrated the effectiveness of our combined peptide and glycan FDR control methods, as they successfully prevented the reporting of any entrapment glycoPSMs in both cases.
