# Supplementary material for: Unraveling the glycosylated immunopeptidome with HLA-Glyco
Source: Nat Commun. 2023 Jun 12;14:3461. doi: 10.1038/s41467-023-39270-2 (PMC10258777; doi:10.1038/s41467-023-39270-2)
Supplement: Supplementary file 2 — Reporting Summary [file 41467_2023_39270_MOESM2_ESM.pdf]

nature portfolio

Corresponding author(s): Alexey I. Nesvizhskii

Last updated by author(s): May 17, 2023

Reporting Summary

Nature Portfolio wishes to improve the reproducibility of the work that we publish. This form provides structure for consistency and transparency in reporting. For further information on Nature Portfolio policies, see our Editorial Policies and the Editorial Policy Checklist.

Statistics

For all statistical analyses, confirm that the following items are present in the figure legend, table legend, main text, or Methods section.

n/a	Confirmed	
☐	☒	The exact sample size (*n*) for each experimental group/condition, given as a discrete number and unit of measurement
☒	☐	A statement on whether measurements were taken from distinct samples or whether the same sample was measured repeatedly
☐	☒	The statistical test(s) used AND whether they are one- or two-sided<br>*Only common tests should be described solely by name; describe more complex techniques in the Methods section.*
☐	☒	A description of all covariates tested
☒	☐	A description of any assumptions or corrections, such as tests of normality and adjustment for multiple comparisons
☐	☒	A full description of the statistical parameters including central tendency (e.g. means) or other basic estimates (e.g. regression coefficient) AND variation (e.g. standard deviation) or associated estimates of uncertainty (e.g. confidence intervals)
☐	☒	For null hypothesis testing, the test statistic (e.g. *F*, *t*, *r*) with confidence intervals, effect sizes, degrees of freedom and *P* value noted<br>*Give P values as exact values whenever suitable.*
☒	☐	For Bayesian analysis, information on the choice of priors and Markov chain Monte Carlo settings
☒	☐	For hierarchical and complex designs, identification of the appropriate level for tests and full reporting of outcomes
☒	☐	Estimates of effect sizes (e.g. Cohen's *d*, Pearson's *r*), indicating how they were calculated

Our web collection on statistics for biologists contains articles on many of the points above.

Software and code

Policy information about availability of computer code

Data collection

No software was used for data collection.

Data analysis

Python anaconda environment for this analysis:
channels:
- conda-forge
- bioconda

dependencies:
- python=3.9
- biopython=1.79
- matplotlib=3.5.1
- plotly=4.14.3
- scikit-learn=1.0.2
- pandas=1.4.1
- pyteomics=4.5.3
- matplotlib-venn=0.11.6
- seaborn=0.11.2
- umap-learn=0.5.2
- hdbscan=0.8.28
- xlrd=2.0.1

- logomaker=0.8

R anaconda environment for this analysis:
channels:
- conda-forge
dependencies:
- r-essentials=4.1
- r-base=4.1.2
- r-devtools=2.4.3

Miscellaneous software:
- FragPipe suite v18.1-build5
- MSFragger v3.5
- Philosopher v4.5.1-RC10
- MoDec v1.1
- NetMHCIIpan v4.1
- MSconvert v3.0.20287

For manuscripts utilizing custom algorithms or software that are central to the research but not yet described in published literature, software must be made available to editors and reviewers. We strongly encourage code deposition in a community repository (e.g. GitHub). See the Nature Portfolio guidelines for submitting code & software for further information.

Data

Policy information about availability of data

All manuscripts must include a data availability statement. This statement should provide the following information, where applicable:

- Accession codes, unique identifiers, or web links for publicly available datasets
- A description of any restrictions on data availability
- For clinical datasets or third party data, please ensure that the statement adheres to our policy

In this study, various databases and repositories were utilized to gather the necessary data for analysis.

The human reviewed protein sequences used in this study are available in the UniProt database under accession code UP000005640.
The Arabidopsis thaliana reviewed protein sequences used in this study are available in the UniProt database under accession code UP000006548.

The proteomic glycosylation data used in this study are available in the GlyGen database (https://www.glygen.org).

The mass spectrometry data used in this study are available in the PRIDE database under accession codes PXD006939 [http://proteomecentral.proteomexchange.org/cgi/GetDataset?ID=PXD006939], PXD020079 [http://proteomecentral.proteomexchange.org/cgi/GetDataset?ID=PXD020079], PXD014017 [http://proteomecentral.proteomexchange.org/cgi/GetDataset?ID=PXD014017], PXD019643 [http://proteomecentral.proteomexchange.org/cgi/GetDataset?ID=PXD019643], PXD020186 [http://proteomecentral.proteomexchange.org/cgi/GetDataset?ID=PXD020186], PXD025877 [http://proteomecentral.proteomexchange.org/cgi/GetDataset?ID=PXD025877], PXD004894 [http://proteomecentral.proteomexchange.org/cgi/GetDataset?ID=PXD004894], PXD013649 [http://proteomecentral.proteomexchange.org/cgi/GetDataset?ID=PXD013649], and PXD012308 [http://proteomecentral.proteomexchange.org/cgi/GetDataset?ID=PXD012308].

The mass spectrometry data used in this study are available in the PRIDE database under accession code PXD005565 [http://proteomecentral.proteomexchange.org/cgi/GetDataset?ID=PXD005565] (yeast enriched N-glycopeptide data).

The processed immunopeptidomics data is available at https://hla-glyco.nesvilab.org.

Research involving human participants, their data, or biological material

Policy information about studies with human participants or human data. See also policy information about sex, gender (identity/presentation), and sexual orientation and race, ethnicity and racism.

Reporting on sex and gender	This information has not been collected.
Reporting on race, ethnicity, or other socially relevant groupings	This information has not been collected.
Population characteristics	This information has not been collected.
Recruitment	This information has not been collected.
Ethics oversight	This information has not been collected.

Note that full information on the approval of the study protocol must also be provided in the manuscript.

Field-specific reporting

Please select the one below that is the best fit for your research. If you are not sure, read the appropriate sections before making your selection.

- [x] Life sciences
- [ ] Behavioural & social sciences
- [ ] Ecological, evolutionary & environmental sciences

For a reference copy of the document with all sections, see nature.com/documents/nr-reporting-summary-flat.pdf

Life sciences study design

All studies must disclose on these points even when the disclosure is negative.

Sample size

We selected a total of eight immunopeptidomic studies (references 31–38) to be included in our analysis. Our selection process prioritized studies that provided a substantial amount of high-resolution mass spectrometry data, aiming to minimize instrument bias.

Through careful curation and annotation of these selected studies, we obtained a collection of 732 distinct HLA class II mass spectrometry samples. These samples encompassed a wide range of data, including 90.8% of HLA-typed data, 80.3% derived from patient tissues, 16.7% from cell lines, and 2.9% from tumor-infiltrating lymphocytes.

The samples we obtained represented up to six different types of cancer located in the brain (meningioma and glioblastoma), skin (melanoma), colon (colorectal), and lung (adenocarcinoma and squamous carcinoma). Additionally, 59% of the samples were non-cancerous and originated from disease-free individuals.

In terms of HLA diversity, our sample collection encompassed up to 72 HLA class II alleles from the three classic genes (DP, DQ, and DR). The number of mass spectrometry samples varied for each allele.

Overall, our sample selection process aimed to provide a comprehensive representation of HLA class II immunopeptidomic data, covering a diverse range of cancer types, non-cancerous samples, and HLA alleles.

Data exclusions

No data were excluded.

Replication

To ensure the reproducibility of the experimental findings, we implemented several measures.

First, we carefully selected a case study of a human B lymphoblastoid cell line (C1R) from Ramarathinam et al. 2011. The HLA-bound peptide purification protocol in this study involved sequential steps with pan anti-class I, class II anti-DP, class II anti-DQ, and class II anti-DR antibodies. This resulted in mass spectrometry samples containing single HLA Class II genes (DP, DQ or DR). This provided four analyses representing the HLA class II alleles DPA102:01/02-DPB104:01 (5 replicates), DQA105:05-DQB103:01 (6 replicates), DRB112:01 (7 replicates), and DRB302:02 (7 replicates), respectively.

We then employed a deconvolution strategy using MoDec for the analysis, a fully probabilistic framework that learns both motifs and preferred binding core position offsets from the peptide sequences themselves. This approach is particularly important when investigating HLA-bound peptides with post-translational modifications like glycosylation, as it avoids the removal of poorly modeled peptides. The deconvolution analysis of these samples consistently showed a similar distribution of glycosylated and non-glycosylated peptides for each HLA allele. Furthermore, statistical analysis using a two-sided Fisher's exact test was performed to compare the HLA-binding cores with and without glycosylation across all 25 replicates. The results demonstrated that all 25 replicates showed an unaltered HLA-binding core in the presence of glycosylation, with P-values greater than 0.05.

In addition to motif deconvolution, we visually verified the position of glycosylation within the HLA binding core for each HLA allele across all mass spectrometry samples in Supplementary Figure 2.

Based on these measures and analyses, the experimental findings related to the glycosylated HLA-associated peptides were deemed reproducible.

Randomization

The mass spectrometry samples were grouped according to their corresponding studies during the allocation process. This means that samples from the same study were analyzed together as a cohesive group. This approach was implemented to ensure a robust modeling of the false discovery rate (FDR) and minimize potential batch effects.

Blinding

The primary focus of this study was to provide a pipeline and comprehensive resource for studying glycosylated HLA-associated peptides. As such, blinding was not necessary or applicable to the objectives of our study.

Reporting for specific materials, systems and methods

We require information from authors about some types of materials, experimental systems and methods used in many studies. Here, indicate whether each material, system or method listed is relevant to your study. If you are not sure if a list item applies to your research, read the appropriate section before selecting a response.

Materials & experimental systems

n/a	Involved in the study
☒	☐ Antibodies
☒	☐ Eukaryotic cell lines
☒	☐ Palaeontology and archaeology
☒	☐ Animals and other organisms
☒	☐ Clinical data
☒	☐ Dual use research of concern
☒	☐ Plants

Methods

n/a	Involved in the study
☒	☐ ChIP-seq
☒	☐ Flow cytometry
☒	☐ MRI-based neuroimaging
