# Supplementary material for: Unraveling the glycosylated immunopeptidome with HLA-Glyco
Source: Nat Commun. 2023 Jun 12;14:3461. doi: 10.1038/s41467-023-39270-2 (PMC10258777; doi:10.1038/s41467-023-39270-2)
Supplement: Supplementary file 3 — Description of Additional Supplementary Files [file 41467_2023_39270_MOESM3_ESM.pdf]

Supplementary Data:
File Name: Supplementary Data 1
Description: entrapment search results.

File Name: Supplementary Data 2
Description: collected mass spectrometry datasets and samples annotation.

File Name: Supplementary Data 3
Description: Glycosylated peptide-spectrum match level information along with GlyGen annotation.

File Name: Supplementary Data 4
Description: Peptide-allele assignment using NetMHCIIpan 4.1 for a fully unsupervised deconvolution.

File Name: Supplementary Data 5
Description: List of the 198 considered glycans, their mass offsets, and types.
